# New Avenues to Chemical Space for Energy Materials by the Molecular Precursor Approach

**DOI:** 10.1002/smsc.202200115

**Published:** 2023-04-12

**Authors:** Suptish Ghosh, Basundhara Dasgupta, Carsten Walter, Prashanth W. Menezes, Matthias Driess

**Affiliations:** ^1^ Department of Chemistry: Metalorganics and Inorganic Materials Technische Universität Berlin Straße des 17. Juni 115, Sekr. C2 10623 Berlin Germany; ^2^ Materials Chemistry Group for Thin Film Catalysis – CatLab Helmholtz-Zentrum Berlin für Materialien und Energie Albert-Einstein-Str. 15 12489 Berlin Germany

**Keywords:** electrocatalysts, hydrogen evolution reaction, molecule-to-material, overall water-splitting reaction, oxygen evolution reaction, reconstruction, single source precursors

## Abstract

The quest to develop efficient electrocatalysts for water‐splitting is still an ongoing challenge. Intense efforts have been dedicated over the years to design effective methods to improve the electrocatalytic performances. In recent times, the single‐source (molecular) precursor (SSP) approach has gained enormous attention from the scientific community as it operates at low temperatures and leads to the formation of unique nanostructured materials, with fine‐tuned chemical and physical properties, resulting in high and stable catalytic activities. Herein, the recent developments in molecule‐to‐material chemistry and their applications toward the oxygen evolution reaction, hydrogen evolution reaction, and overall water‐splitting are summarized. Furthermore, the review focuses on understanding the reconstruction process of the SSP‐derived materials and the adopted techniques (in situ and ex situ) to obtain insights into the active structures for catalysis. The future possibilities of applying these materials for value‐added organic electro‐oxidation/reduction reactions are also explored.

## Graphical abstract

The recent developments in single‐source‐precursor‐derived materials synthesis for water‐splitting applications and their reconstructions under electrochemical conditions are summarized.

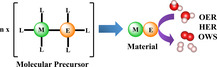

## 1 Introduction

The development of sustainable technologies based on renewable resources (solar, wind, etc.) is the key to mitigate excessive carbon dioxide (CO_2_) emissions, and for achieving a carbon‐neutral economy.^[^ ^1^ ^]^ However, the intermittent nature of solar and wind energies prohibits their direct and extensive real‐life implementation. In this regard, molecular hydrogen (H_2_) offers the highest gravimetric energy content (≈143 MJ kg^−1^), which is three times higher as compared to fossil‐fuel‐derived energy (diesel or gasoline ≈47 MJ kg^−1^).^[^ ^2^ ^]^ Therefore, the production and storage of green hydrogen gas is the key to fulfill the global energy demands. Among many available technologies to produce H_2_, electrolysis of water, driven by renewable energy sources, is one of the most sustainable approaches.^[^ ^3^ ^]^ This technology is also highly attractive due to its large scalability opportunities, and its environmentally benign nature.^[^ 4, 5 ^]^

A recent study showed that the demand for H_2_ energy is increasing rapidly, with a predicted requirement of 600 Mt in the year 2050, in comparison with <100 Mt in recent times.^[^ ^6^ ^]^ Therefore, to meet this demand, various water electrolyzer cell (WEC) technologies have been developed over the years.^[^ ^7^ ^]^ Considering the pH of the electrolyte, for the alkaline medium, an alkaline electrolyzer cell (AEC), and for the acidic medium, a polymer electrolyte membrane (PEM) has been implemented intensively to produce H_2_ from H_2_O.^[^ 8, 9 ^]^ However, each electrolyzer type comes with its own merits and demerits; PEM offers higher efficiencies but requires scarce noble metals and noble‐metal‐oxide‐based catalysts,^[^ ^9^ ^]^ whereas, AEC runs on earth‐abundant transition‐metal‐based catalysts for longer times, as compared to PEM.^[^ ^10^ ^]^

To facilitate the electrolysis of water with high efficiencies, the development of competent electrocatalysts is of utmost importance. To date, state‐of‐the‐art catalysts RuO_*x*_ and IrO_*x*_ are known to showcase the best performance for oxygen evolution reaction (OER) and Pt for hydrogen evolution reaction (HER).^[^ 11, 12 ^]^ Considering the price and the non‐abundant nature of these metals, intense efforts have been put into designing cost‐effective electrocatalysts based on transition‐metal (TM) oxides/hydroxides/(oxy)hydroxides,^[^ 13, 14, 15, 16, 17 ^]^ chalcogenides,^[^ 18, 19, 20 ^]^ pnictides,^[^ ^21^ ^]^ phosphides,^[^ ^22^ ^]^ borides,^[^ 23, 24 ^]^ borophosphates,^[^ 25, 26 ^]^ carbides,^[^ ^27^ ^]^ alloys,^[^ ^28^ ^]^ intermetallics,^[^ 29, 30, 31, 32 ^]^ and their heterostructures; as well as ligand‐assisted metal–organic frameworks (MOFs),^[^ 33, 34, 35 ^]^ covalent organic frameworks (COFs),^[^ 36, 37 ^]^ and metal oxalates^[^ 38, 39 ^]^ for both OER^[^ 40, 41 ^]^ and HER^[^ ^42^ ^]^ reactions. Most of these electrocatalysts show promising results in comparison to the benchmark noble‐metal‐based state‐of‐the‐art catalysts.

To prepare this huge variety of materials, several synthetic methods have been adopted, starting from the high‐temperature solid‐state methods, such as annealing, pyrolysis, calcination, thermolysis, and microwave‐assisted synthesis, to low‐temperature wet chemical precipitate approaches.^[^ ^43^ ^]^ However, most of these synthetic approaches often result in poor control over the morphology, composition, as well as chemical and electronic properties of the materials, leading to poor activities. Considering this, one of the most rationalized approaches to produce well‐controlled, catalytically efficient materials is from well‐designed, suitable single‐source molecular precursors (SSPs).^[^ ^43^ ^]^ Such molecule‐to‐material conversation can be easily achieved by using low‐temperature liquid (and)/gas‐phase transformation approaches like co‐precipitation, condensation, micro‐emulsion, colloidal, and hydrothermal pathways. Furthermore, the serendipity of the SSP approach lies in the facile formation of a single crystalline phase and the unique physical and chemical properties of the obtained material.^[^ 14, 21, 44, 45, 46 ^]^ However, this approach could lead to a substantial carbon content in the finally formed materials, due to the presence of the organic ligand in the precursor and this should always be considered. The high carbon content from the organic ligand transformation into a carbon matrix support often has a desired effect and could enhance the catalytic activity due to the improved conductivity of the composite material. However, the carbon of SSP‐derived material usually forms amorphous carbon which is nonconducting, and, such carbon by‐products remain as a separate entity rather than intimately mixing with metals.^[^ ^47^ ^]^ Furthermore, the cost of the precursor can limit further practical applications.^[^ ^43^ ^]^ Nevertheless, such low‐temperature molecular precursor approaches have been proven to be beneficial to improve the activity and stability of electrocatalysts for OER, HER, and overall water‐splitting (OWS) reactions.^[^ 14, 21, 44, 45, 48 ^]^

In this review, we will summarize the recent developments in SSP‐based materials and their transformation pathways for the formation of distinct active structures/phases under electrochemical OER, HER, and OWS conditions. Furthermore, we will discuss the adopted in situ and ex situ characterization techniques to reveal the true active structures/phases of these materials under electrochemical conditions. In the end, we will present the possibility of applying these SSP‐derived materials toward value‐added organic oxidation/reduction reactions.

## 2 Fundamentals of Water‐Splitting and the Catalyst/(Pre)Catalyst Reconstruction

Electricity‐driven water‐splitting consists of two half‐cell reactions, wherein the reduction process occurs at the cathode to produce hydrogen gas (HER), and the oxidation process occurs at the anode to produce oxygen gas (OER).^[^ 49, 50 ^]^ Although both OER and HER are energy‐intensive reactions, however, it is the OER that is considered as the bottleneck for the OWS reaction, due to the involvement of multi‐proton‐electron‐coupled transfer.^[^ ^51^ ^]^ However, OER is crucial to supply the necessary electrons and protons to produce H_2_ at the counterpart.^[^ 52, 53, 54 ^]^ The theoretical potential required for OER and HER are 1.23 and 0 V, respectively (at 25 °C in 1 atm pressure) to derive the OWS reaction.^[^ 55, 56 ^]^ Due to the sluggish kinetics of OER and HER, along with cell and catalyst resistances, in practice, an extra potential is required to drive these reactions to attain a set current density, which is called the overpotential (*η*).^[^ ^57^ ^]^ Therefore, the overpotential, along with reaction kinetics and stability, are important matrices to judge/screen the efficiency of electrocatalysts for OER and HER, which have been extensively described in previous literature.^[^ 19, 58, 59, 60 ^]^

One of the imperative assets that we try to understand in this review is about the types of material reconstruction processes, and their role in activity enhancement. Under an electrochemical environment, most of the TM‐based materials mentioned earlier behave as (pre)catalysts and are transformed into active catalysts to drive the OER and HER.^[^ 15, 26, 36, 37, 42 ^]^ Currently, four types of reconstruction pathways have been observed, which are 1) no‐reconstruction, 2) near‐surface, 3) partial (core–shell), and 4) complete reconstructions.^[^ 52, 61, 62 ^]^ The nature of such transformation is completely unpredictable and depends on the kind of material. However, such transformations are often proven beneficial to modify/improve the catalyst's intrinsic and extensive properties.^[^ ^52^ ^]^ One classical example of this is Raney nickel, which is intensively used in industries, where Al is used as a sacrificial element to create a porous Ni–skeletal catalyst, with a high surface area.^[^ ^63^ ^]^ Nonetheless, all the aforementioned reconstruction processes depend on several factors, such as temperature and pH of the medium, applied potential, crystal structure of the material, composition, morphology, and electronic properties.^[^ 15, 18, 64 ^]^ In general, complete reconstruction is more beneficial to achieve better activities, since it allows greater penetration of the electrolyte into the bulk of the catalyst, thus accelerating the reaction kinetics.^[^ ^45^ ^]^ Some recent studies also indicated that high working temperatures, electrolyte concentrations, and electrolyte medium (saline water, buffer medium) can also trigger the reconstruction process.^[^ 15, 65, 66, 67, 68 ^]^ In an alkaline medium, leaching exposes more bulk active sites, and similarly, in an acidic medium, leaching or corrosion is dominant, which leads to a more surface‐rich metal site.^[^ ^69^ ^]^ Additionally, the working potential also has a direct impact on the degree of reconstruction, which has already been discussed in previous literature.^[^ ^70^ ^]^

## 3 Single‐Source (Molecular) Precursor

The SSPs are usually organometallic complexes, containing all the desired choices of metals and nonmetals with a soft ligand backbone.^[^ ^71^ ^]^ This approach is advantageous to rationalize material synthesis and ensures well‐defined stoichiometry and homogeneity when compared to multisource approaches. It also leads to the formation of nanosized particles, with high surface areas. It reduces the crystallization temperature required for material synthesis and usually results in the formation of pure phases.^[^ 47, 72 ^]^ The preexisting bonds between the elements in the SSP also help to lower the nucleation barrier.^[^ ^43^ ^]^

## 4 Synthetic Methods to Derive Materials from Molecular SSPs

**Figure 1:**
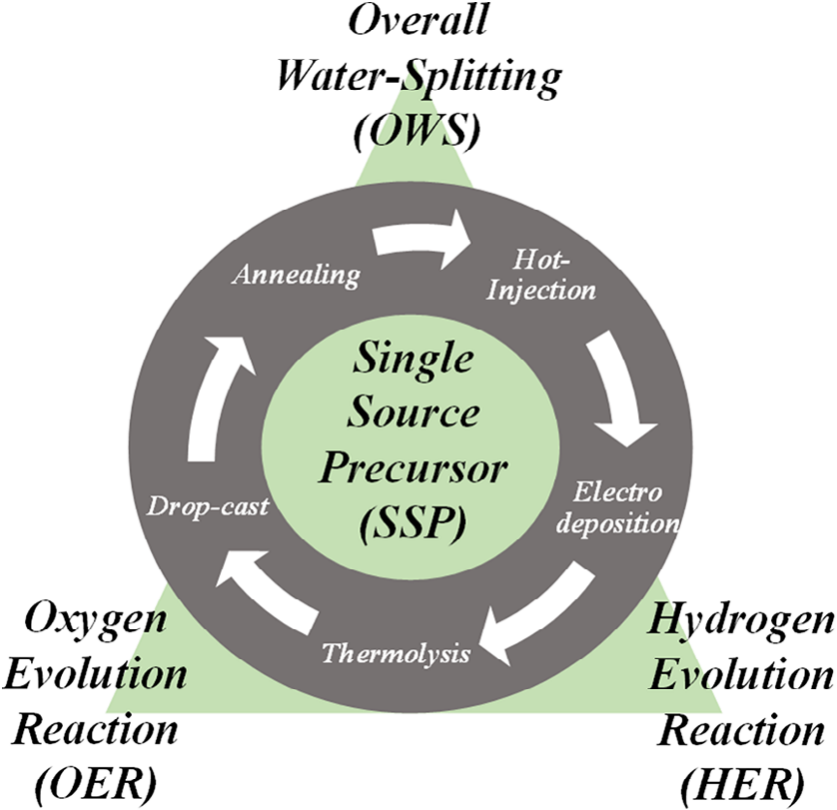
Schematic representation of different types of adopted synthetic techniques to derive energy materials from single‐source precursor (SSP) molecule.

In this review, we mainly focus on such nanomaterials derived from SSPs via a low‐temperature approach. As shown in **Figure** **1**, one of the most adopted techniques is the hot‐injection method, where the molecular complex is decomposed in a hot solvent to obtain the desired product.^[^ 14, 45, 48 ^]^ Additionally, solvent‐free annealing of molecular precursors under a gaseous (or inert) atmosphere is also a well‐used strategy to obtain highly defined materials.^[^ 44, 73, 74 ^]^ The low‐temperature thermolysis is also widely used to produce desired metal oxides from a suitable molecular precursor.^[^ ^72^ ^]^ Most recently, electrodeposition is also adopted to fabricate molecular precursors directly on the substrate for electrochemical studies.^[^ 75, 76 ^]^ Apart from these traditional methods, several advanced techniques have been used to produce nanomaterials, namely, thermolysis, solvolysis, and drop‐casting.^[^ 77, 78, 79 ^]^

It is worth mentioning here that MOFs is a different class of polymeric inorganic material, which is also analogous to SSP.^[^ ^80^ ^]^ MOF materials have been used extensively as promising electrocatalysts for OER/HER/OWS in the last few years.^[^ ^33^ ^]^ However, as several reviews already exist that solely focus on MOF‐based materials for electrocatalytic application, we have excluded these materials from this review.^[^ 81, 82 ^]^

## 5 SSP‐Derived Electrocatalysts Toward OER, HER, and OWS

**Figure 2:**
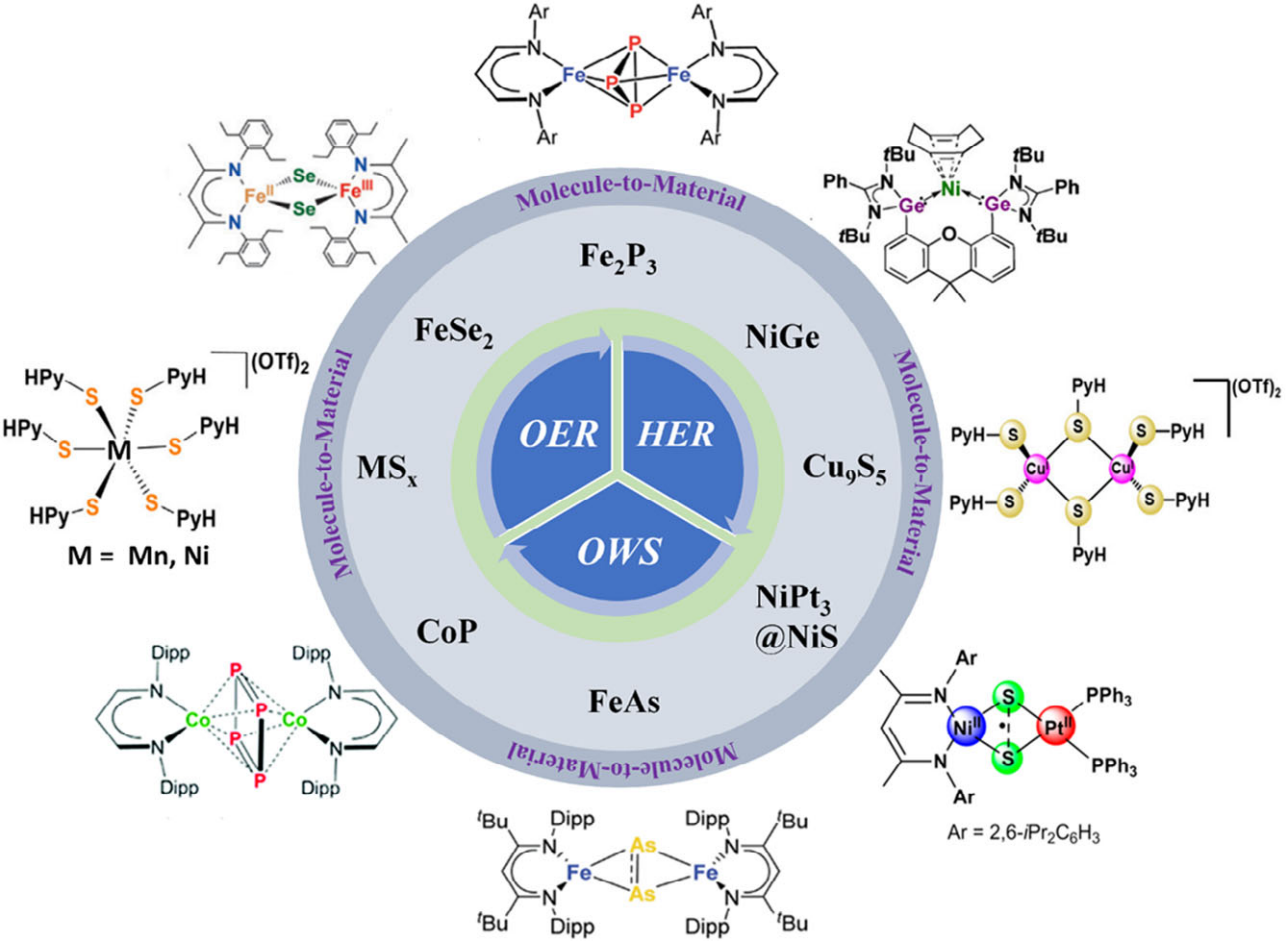
Molecular structure of SSPs and their corresponding energy materials, prepared via low‐temperature approach.

As mentioned earlier, the obtained novel materials from the SSPs are attractive choice electrocatalysts for OER, HER, and OWS (**Figure** **2**).^[^ 14, 21, 28, 44, 45, 83, 84 ^]^ Its advantage to fine‐tune the structure, morphology, and particle size at relatively mild temperatures and even design material on an atomic scale to achieve compositions that would not be accessible via traditional methods has increased the interest in the last years as a benign design method for various electrocatalytic applications.^[^ 72, 85 ^]^ A facile approach is the direct deposition and decomposition of the molecular precursor onto the substrate used as the working electrode in water‐splitting. For example, the group of Ziani prepared phthalocyanine (CoPc) films with a varying thickness on different substrates and annealed the films at 400 °C in air to form oxide nanoparticles (NPs). The highly transparent films of cobalt oxide demonstrated a similar performance for OER when compared with other cobalt‐based catalysts used to functionalize the surface of a BiVO_4_/WO_3_ photoanode.^[^ ^86^ ^]^ In a recent study by Swierk and Don Tilley from 2018, a Co[N(SiMe_3_)_2_]_2_ precursor was prepared and directly deposited onto indium‐tin‐oxide (ITO) electrodes from toluene in a glove box. The precursor was allowed to graft for 1 h and dried with a stream of nitrogen, followed by heating at 300 °C in air to decompose the precursor. At low loadings, a cobalt (II) species (SS–Co) was formed predominantly consisting of single, isolated atoms (SS–Co), while small clusters of Co_3_O_4_ were formed at higher loadings. The OER activity for the SS–Co species was observed to be lower than for the Co_3_O_4_ clusters, explained by the mononuclear mechanism in SS–Co, which is considered to be disfavored.^[^ ^87^ ^]^ Already early studies by Stracke and Finke in 2011 have shown that molecular precursors, at least partially, decompose at mild conditions during the electrochemical treatment on a substrate. Their Co‐containing polyoxometalate in [Co_4_(H_2_O)_2_(PW_9_O_34_)_2_]^10−^ partially decomposed, releasing Co^II^ to form a well‐precedented active CoO_*x*_ under oxidizing conditions.^[^ ^88^ ^]^ Though later studies in 2013 suggested that the OER activity cannot be completely explained by the formation of CoO_*x*_, it demonstrated a facile molecule‐to‐material approach via electrophoretic decomposition.^[^ ^89^ ^]^ In the same year, Reisner et al. reported the formation of CoO_*x*_ as efficient OER electrocatalysts from the nanosized TiCo cages [Ti_12_O_15_(O^*i*^Pr)_17_]^+^[(CoBr)_6_Ti_15_O_24_(O^*i*^Pr)_18_(Br)]^−^ and [(CoI)Ti_11_O_14_(O^*i*^Pr)_17_] as precursors that were drop‐casted onto the electrodes in situ forming the active material at applied positive potential.^[^ ^90^ ^]^ The group later used a [Ti_2_(OEt)_9_(NiCl)]_2_ (TiNi_SSP_) and a [Ti_4_O(OEt)_15_(CoCl)] (TiCo_SSP_) complex directly grafted onto an fluorine‐doped tin oxide substrate that transformed into NiO_*x*_ and CoO_*x*_ species acting as oxygen evolving centers during anodic current and was also applied for photoelectrochemical water‐splitting.^[^ ^91^ ^]^ Very recently, they reported the use of [Zr_4_O(EtO)_15_Co^II^Cl], [Zr_4_(O)_2_(EtO)_16_Fe_2_^III^Cl_2_], and [Zr_4_(O)_2_(EtO)_18_Cu_4_^II^Cl_4_] complexes as SSPs for (photo)electrochemical applications using the same method.^[^ ^92^ ^]^ A bimetallic Co–Mo alkoxide [Co_3_Mo_4_O_10_(OCH_3_)_10_(dmf)_4_] was used by Kuznetsov and his group as a precursor to form a Co_cat_ for efficient OER electrocatalysis in 0.1 m sodium borate buffer (NaBi, pH 9.2) and 1 m NaOH. The drop‐casted precursor decomposed at an applied positive potential where Mo was completely lost from the films giving rise to high surface area films identified as cobalt oxo‐hydroxide.^[^ ^93^ ^]^ Spiccia and his group received similar results for their electrodeposited [Ni(en)_3_]Cl_2_ (en = 1,2‐diaminoethane) precursor to form a NiO_*x*_‐en film from a 0.10 m borate buffer (NaBi) solution (pH = 9.2). The NiO_*x*_‐en films were more homogenous and had a higher electroactive surface area than films prepared from a simple Ni nitrate precursor, leading to more robust films with higher catalytic activity underlining the importance of the choice of molecular precursors.^[^ ^79^ ^]^ Later, Chen et al. used a tris(hydroxymethyl) aminomethane (Tris) ligand to attain a Ni‐Tris complex, in situ formed in a borate buffer solution, that was transformed into a NiO_*x*_, giving an ultrathin film with high‐electrocatalytic performance for OER in borate buffer solution.^[^ ^94^ ^]^ The buffer‐free approach to electrodeposit NiO_*x*_ in alkaline conditions by the group of Meyer further underlined the significance of using SSPs for material preparation. The pyridinedimethanol ligand (HL) from the molecular precursor complexes [Ni^II^_4_(HL)_4_(OAc)_4_] and [Ni^II^(H_2_L)_2_](NO_3_)_2_ was described as an important factor in the complex‐to‐nickel hydroxide conversion under basic conditions, to prepare NiO_*x*_ films with excellent electrocatalytic activity toward electrocatalytic OER in alkaline conditions.^[^ ^76^ ^]^ The electrophoretic decomposition of cubane‐like SSPs at an applied potential was extended by Yano and Agapie to well‐defined bimetallic [CoMn_3_O_4_] and [NiMn_3_O_4_] cubane precursors, in which they observed the formation of mixed metal MnO_*x*_ films with novel composition for OER electrocatalysts.^[^ ^95^ ^]^ All the conducted studies show a benign SSP method to access OER electrocatalysts as well‐defined films and structures, that were not accessible with traditional methods using metal salts. It illustrates how the choice of discrete precursors fine‐tunes the structure and composition of the resulting solid catalytic materials.^[^ 94, 96 ^]^ In extension to obtained oxide materials, the group of Savéant observed the formation of Co nanoparticles from the electrophoretic decomposition of the Co[(diphenylglyoxime)_3_(BF)_2_]BF_4_ complex at reducing potentials. The synthesized nanoparticles showed remarkable activity toward HER in water at pH 7 at low overpotential.^[^ ^97^ ^]^ Liaw et al. reported the electrodeposition of film with a combination of iron oxide, metallic iron, and carbonate from molecular dinitrosyl iron complex (DNIC) {Fe(NO)_2_}^9^ [(Me_6_tren)Fe(NO)_2_]^+^ (Me_6_tren = tris[2‐(dimethylamino)ethyl]amine). The amorphous FeO_*x*_–Fe–CO_3_^2−^ material showed enhanced activity for HER, which was oxidized into an iron oxide/hydroxide species during electrocatalytic OER.^[^ ^98^ ^]^ Lately, Zhang and Cao reported the formation of NiSe nanospheres using nickel selenite complex [{Ni(TMEDA)SeO_3_}_2_] to electrodeposit the material on carbon cloth. The NiSe–TMEDA/CC demonstrated bifunctional OER and HER activity in the alkaline solution for water‐splitting with low overpotentials.^[^ ^99^ ^]^ Another very benign technique to form scalable binder‐free thin films directly onto an electrode substrate is the chemical vapor deposition (CVD) method. FeMnP was deposited onto graphene‐protected nickel foam (GNF) by metal–organic CVD (MOCVD) using the FeMn(CO)_8_(*μ*‐PH_2_) as SSP and applied the material as both the anode and the cathode in overall water‐splitting in 0.1 m KOH. The uniform films of FeMnP/GNF showed remarkable electrochemical activity while the phase‐pure FeMnP species was oxidized during OER into FeO_*x*_ and MnO_*x*_ as active species. However, during HER, the phosphide phase remained and synergistic effects between Mn and Fe were found to be responsible for its magnificent performance.^[^ ^100^ ^]^ They also deposited the FeMnP onto rutile TiO_2_ substrate and used the formed electrode as photoanode and for electrochemical OER, and a (Mn_1–*x*_Fe_*x*_)OOH/FeMnP/TiO_2_ core–shell structure was formed during the oxidizing conditions, leading to high efficiency and stability.^[^ ^101^ ^]^ In a comparative study, a series of iron phosphides, Fe_*x*_P (*x* = 1–3) were prepared using different SSPs by the CVD process. The FeP films were prepared using either Fe(CO)_4_P^*t*^BuH_2_ or {Fe(CO)_4_P(H)^*t*^Bu}_2_ as precursors while Fe_2_P films were made from the Fe(CO)_4_PH_3_ complex, and Fe_3_P films using the H_2_Fe_3_(CO)_9_P^*t*^Bu complex. The materials were tested for HER activity following the trend Fe_3_P > Fe_2_P > FeP, correlating higher metal content with better HER performance. Therefore, the activity trend was explained with hydrogen binding favoring the iron‐rich surfaces of Fe_3_P and Fe_2_P over the iron‐poor FeP.^[^ ^102^ ^]^ The CVD method was also used to prepare a metal‐free electrocatalyst material for OER. Leardini and his group used methylamine borane (BH_3_NH_2_CH_3_) as SSP for their approach to grow B–C–N layers on Ti substrates by varying the growth time to obtain a Ti‐BCN and TiCN–BCN species with ratios of B:C:N = 0.3:1.0:0.4 and 0.6:1.0:0.9, respectively. Although both the samples showed lower activity than a Pt reference sample, the B–N‐rich sample performed best, which was explained by the highly heterogeneous bonding scheme in the TiCN–BCN sample that enhanced the OER activity.^[^ ^103^ ^]^ Additionally, the graphitic carbon nitride (g‐C_3_N_4_) is known to serve as an excellent precursor for the synthesis of metal‐free nitrogen‐doped carbon‐based catalysts for photo‐ or electrochemical water‐splitting catalysts as well.^[^ ^104^ ^]^

**Figure 3:**
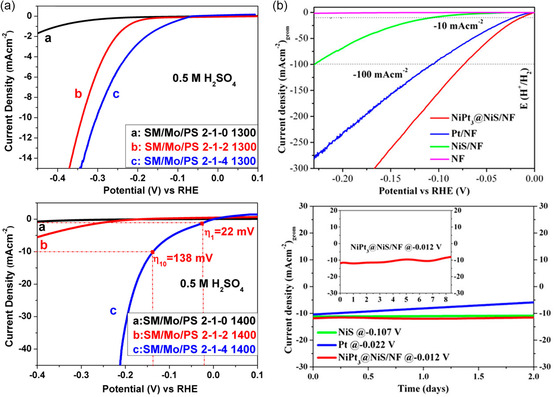
Electrochemical hydrogen evolution reaction (HER) activity of SSP‐derived electrocatalysts. a) Polarization curves (*i*R corrected) of samples derived from the Nowotny phase, Mo_3+2*x*_Si_3_C_0.6_ in the acidic medium (0.5 m H_2_SO_4_). Reproduced with permission.^[^ ^114^ ^]^ Copyright 2019, Wiley‐VCH. b) Linear sweep voltammetry polarization curves of activity assessment (top) and long‐run chronoamperometry (CA) test in alkaline medium (1 m KOH) (inset shows over 8 days of CA for NiPt_3_@NiS/nickel foam (NF)) (bottom). Reproduced with permission.^[^ ^28^ ^]^ Copyright 2019, American Chemical Society.

The precursor can also be transformed first into the desired material before deposition onto a substrate and further application as the working electrode in electrochemical water‐splitting. A very mild approach was described by the group of Kuznetsov, they used the [Ni_2_Mo_2_O_4_(OCH_3_)_8_(CH_3_OH)_2_(*N*,*N*‐dimethylformamide)_2_] complex (Ni_2_Mo_2_) dissolved in methanol to drop‐cast a film onto the electrode substrate. The solvent was evaporated at room temperature and the film was hydrolyzed with a water droplet followed by etching with NaOH solution resulting in a porous film with highly homogeneously distributed Ni, Mo, and O. The active species for the electrochemical OER was identified as NiO_*x*_/NiOOH with excellent activity.^[^ ^105^ ^]^ Solid‐state thermolysis has been shown to be a simple and effective route to transform SSPs into materials. A convenient method was used by Shevchenko et al. to synthesize transition‐metal phosphides by using a bis(diphenylphosphine) as a ligand in their organometallic complex. The attained CoP, NiP, and FeP exhibited low OER overpotentials in alkaline media with CoP showing favorable kinetics for the OER activity compared to the Fe and Ni phosphide catalysts.^[^ ^106^ ^]^ A metallic cobalt in a N‐doped carbon shell (Co@NC), with high OER activity, has been obtained in a very similar way using a [Co^II^Cl_3_N(CN)_2_]·3CH_3_OH complex and the ligand as a precursor for a carbon matrix support.^[^ ^107^ ^]^ Based on this concept, Barman and Nanda et al. reported in 2016, a novel SSP approach to transition‐metal‐rich sulfides Co_9_S_8_ and Ni_3_S_2_ phases with exceptional OER performance in an alkaline medium via a metal–chelate complex tris(ethylenediamine) metal (II) sulfate) as the SSP. The metal sulfide nanostructures were embedded in a nitrogen‐doped carbon (NC) framework, resulting in Co_9_S_8_@NC and Ni_3_S_2_@NC, where the ethylenediamine served as a precursor for carbon nitride matrix during pyrolysis.^[^ ^74^ ^]^ Not long ago, Akram and Kahn used various alkyl xanthate complexes of cobalt (alkyl = ethyl, hexyl, octyl) to synthesize nanoparticles of cobalt sulfide (CoS) by a solventless route. The xanthate complexes of varying alkyl chain lengths affected the morphology of the nanoparticles. After pyrolysis, the agglomeration of the particles decreased with the increased number of carbons in the chain, causing more steric hindrance to the particles in proximity. It was observed that for HER and OER, the efficiency was affected by the size of the nanoparticles, wherein a smaller size resulted in enhanced electrocatalytic performance.^[^ ^108^ ^]^ In 2017, the group of Pinna reported the formation of nickel phosphide nanoparticles with various compositions (Ni_12_P_5_, Ni_12_P_5_–Ni_2_P, and Ni_2_P) using layered nickel phenylphosphonate (NiPh) or methylphosphonate (NiMe) as SSPs. The attained Ni_12_P_5_–Ni_2_P and Ni_2_P nanoparticles were successfully applied to efficiently catalyze the HER, exhibiting high activity and long‐term stability. Furthermore, they used the same method to synthesize Co_2_P and CoP phases to demonstrate the versatility of this approach to form various phosphide‐based materials for more electrochemical energy‐conversion applications.^[^ ^109^ ^]^ Zhu and co‐workers presented an in situ coupling approach for a new highly efficient and durable cobalt‐based alloy electrocatalyst. The FeCo–Co_4_N/N–C for electrocatalytic OER and oxygen reduction reaction (ORR) was synthesized from a nanoporous phthalocyanine‐based organic framework pyrolyzed under inert condition. The diverse active sites of FeCo and Co_4_N nanoparticles were key elements that led to the high‐electrocatalytic bifunctional activity.^[^ ^73^ ^]^ A year later, a similar approach was used by Liao et al. for the synthesis of mono‐(Fe or Ni) and bimetallic (Fe–Ni) heteroatoms/carbon catalysts using the covalent organic polymers as SSPs. The derived highly efficient FeNi–composite materials were applied as electrocatalysts for ORR and OER, surpassing the activity of the monometallic composites of Ni and Fe.^[^ ^110^ ^]^ At the same time, Chen et al. prepared a CoFe_*x*_–CoFe_2_O_4_/N‐doped carbon nanocomposite by pyrolysis of {[Co(H_2_O)_6_][Fe(Hedta)Cl]_2_·4H_2_O]} used as SSP.^[^ ^111^ ^]^ The hierarchical porous structure of the CoFe_*x*_–CoFe_2_O_4_/N‐doped carbon showed high performance in alkaline HER and OER with small overpotentials, which was attributed to the high electronic conductivity, large surface area, and strong tolerance to the alkaline environment.^[^ ^111^ ^]^ Very recently, the group of Nanda synthesized alloys of Co encapsulated within NC polyhedra along with short carbon nanotubes (CNTs) using the MOF ZIF‐67 combined with corresponding metal salts as SSP. The derived MCo@NC (M = Pt, Pd, Ru) materials demonstrated bifunctional catalytic activity toward HER and OER in alkaline media.^[^ ^112^ ^]^ Various metallic composites, for example, FeCo@NC FeNi@NC, CoNi@NC, Fe@NC, Co@NC, and Ni@NC were attained in a one‐pot procedure by mixing the corresponding metal salts (nitrates) with tannic acid and polyethylenimine (PEI) to form a solid tannic acid–PEI–metal precursor, that was pyrolyzed in an argon atmosphere at 900 °C to access the nanoparticle composite materials. The formed electrocatalysts showed high OER activity and durability in 1 m KOH with FeNi@NC.^[^ ^113^ ^]^ In the meantime, the group of Riedel prepared a ternary Nowotny phase, with the composition Mo_3+2*x*_Si_3_C_0.6_ (*x* = 0.9–0.764), for electrochemical water‐splitting. The Nowotny phase was embedded in a porous SiC/C nanocomposite matrix that was synthesized via the SSP approach, by reacting allylhydridopolycarbosilane (AHPCS) with MoO_2_(acac)_2_, followed by thermal treatment at comparatively high temperature in inert conditions but necessary to form the nanocrystalline Mo_3+2*x*_Si_3_C_0.6_ carbide material with the improved surface area when compared to samples synthesized at a lower temperature. The electrocatalytic activity of the attained Mo_3+2*x*_Si_3_C_0.6_/C/SiC nanocomposite toward acidic HER exceeded that of most Mo‐based electrocatalysts and showed high stability during long‐term measurements (**Figure** **3**a).^[^ ^114^ ^]^ They used the same method, but with tungsten hexachloride (WCl_6_) instead of MoO_2_(acac)_2_ to also obtain W‐containing SiC‐based nanocomposites and used the material as HER electrode for electrocatalytic studies.^[^ ^115^ ^]^ Often for this class of material, higher decomposition temperatures are needed to proceed with the carbothermal reduction, with the carbon source acting as the reducing agent, to reduce metal oxides and form the metal carbides. The pomegranate‐like Mo_2_C@C nanospheres which were highly active for electrocatalytic HER were derived from phosphomolybdic acid (H_3_PMo_12_O_40_·*n*H_2_O) that needed to be pyrolyzed at least at 700 °C to start the carbothermal reduction.^[^ ^116^ ^]^ A similar approach at comparably high temperatures was used to synthesize manganese–vanadium oxide/carbide nanoparticles embedded in multi‐walled carbon nanotubes (MWCNTs) as very active electrocatalysts for OER, ORR, and chlorine evolution. The mixture of Mn_7_C_3_, V_8_C_7_, and MnO nanoparticles attached to the MWCNTs surface received from a [Mn_4_V_4_O_17_(OAc)_3_]^3−^ precursor could only be achieved at 900 °C, lower temperatures led to solely oxide materials without carbide species and significantly lower surface area, by the power of 10, compared to the carbide material.^[^ ^117^ ^]^ The same [Mn_4_V_4_O_17_(OAc)_3_]^3−^ precursor was used by the group in a hydrothermal approach to synthesize a nanostructured MnVO_*x*_ composite on a nitrogen‐doped reduced graphene oxide (N‐rGO). Decomposition at 440 °C led to the formation of spherical Mn_1.5_V_1.5_O_4_ nanoparticles, while decomposition at 900 °C yielded a Mn_2_V_2_O_7_ phase, both with promising OER activity and long‐term stability even under harsh electrocatalytic conditions in alkaline media. They observed that raising the decomposition temperature from 440 to 900 °C improved the electronic properties of their carbon matrix, since more carbon has been reduced by calcination at higher temperatures to attain rGO. Further, they stated that higher temperatures can be used to tune the chemical environment of nitrogen in N‐doped rGO and additionally improve the electronic properties, without affecting the size of the metal–oxide nanoparticles.^[^ ^118^ ^]^ A year later, Pan et al. used the hydrothermal method to access transition‐metal carbides (TMCs) in a MOF matrix. The hydrothermally prepared CuWO_4_ precursor on a zeolitic imidazolate framework (ZIF), CuWO_4_@ZIF‐67 was pyrolyzed in an argon atmosphere to achieve the nitrogen‐doped carbon‐hosted polymetallic carbide (NC@Cu–Co–W–C) material. The nanoparticle composite material was highly active for OER, HER, and overall water‐splitting which was associated with the positive influence of the local electronic structure reconfiguration that promotes high‐valence‐state Co^3+^ and low‐valence‐state Cu and W species to enhance the electrochemical activity of the catalyst.^[^ ^119^ ^]^ A milder approach to TMCs was described by Strouse's group lately using Prussian blue analogues (PBA) as SSPs. The prepared Co_2_C and Ni_3_C carbide phases were prepared from K[CoCo(CN)_6_]·*x*H_2_O; Ni[Ni(CN)_4_] by dissolving the M‐PBA in octadecylamine and heating the system up to 350 °C retrieving the precipitate by centrifugation and washing. For Fe_3_C, the procedure remained the same only that a commercially available Fe‐PBA was used. The electrocatalysts were tested for alkaline OER and acidic HER but only Ni_3_C showed good activity as an amphoteric electrocatalyst for water‐splitting but remained less active compared to literature ref. [120] Not long ago, Revaprasadu et al. in 2021 used a molecular precursor of bismuth (tris(selenobenzoato)bismuth(III), [Bi(SeOCPh)_3_]), to prepare selectively Bi or Bi_2_Se_3_ nanosheets. The Bi_2_Se_3_ nanosheets were received by injecting the [Bi(SeOCPh)_3_] complex dispersed in 1‐octadecene, into oleylamine preheated at 200 °C while pure Bi was attained when trioctylphosphine was used instead of 1‐octadecene. Using the CVD approach led exclusively to a Bi_2_Se_3_ thin films. The Bi and Bi_2_Se_3_ nanosheets were tested for overall water‐splitting application, where Bi showed lower OER but better HER performance. Furthermore, Bi_2_Se_3_ nanosheets exhibited photoelectrocatalytic activity, indicating a p‐type behavior.^[^ ^78^ ^]^ Improving their SSP method, to obtain nickel sulfide and phosphide particles, three different nickel(II) dithiophosphonate complexes of the type [Ni{S_2_P(OR)(4‐C_6_H_4_OMe)}_2_] [R = H (**1**), C_3_H_7_ (**2**)] and [Ni{S_2_P(OR)(4‐C_6_H_4_OEt}_2_] [R = (C_6_H_5_)_2_CH (**3**)] were employed. The solventless decomposition of **1** and **2** led to hexagonal NiS while surfactant/solvent reaction with **1**, **2**, or **3** at 300 °C in varying mixtures of tri‐octylphosphine oxide (TOPO), hexadecylamine (HAD), and tri‐*n*‐octylphosphine (TOP) led to the formation of rhombohedral NiS, and hexagonal Ni_2_P and Ni_5_P_4_. While the nickel phosphide species showed better performance during electrochemical OER, the hexagonal nickel sulfide species was more active during HER.^[^ ^121^ ^]^ Similarly, Pd_6_P nanospheres were attained in a one‐pot thermolysis at 250 °C in a 1:1 solution of oleylamine and oleic acid. The resulting nanospheres were immobilized onto graphene oxide (GO) at room temperature in ethanol to from the GO–Pd_6_P composite. During electrochemical HER, Pd partially leached from the structure and created vacancies within Pd_6_P causing changes in the electronic structure of the material leading to an enhanced activity.^[^ ^122^ ^]^ Muddassir et al. reported a convenient route to ternary copper tungsten sulfide nanoparticles applied for alkaline and acidic OER, and HER. They used a WCu_2_S_4_(PPh_3_)_3_ complex, that was dissolved in octadecyl amine and heated to 350 °C to obtain the Cu_2_WS_4_ nanoparticles that performed better in OER than in HER when comparing the current density, but had a better Tafel slope and onset potential for HER compared to the OER activity parameters.^[^ ^123^ ^]^

Our group has also synthesized and investigated various molecular precursors with a broad variety of elemental compositions to access new functional energy materials for application in OER, HER, and OWS. Most recently, the combination of NiPt_3_@NiS heterostructure from novel subsulfido‐bridged heterobimetallic nickel–platinum precursor was applied toward an alkaline HER reaction (Figure 3b). Notably, the reported overpotential outmatched the state‐of‐the‐art (Pt) HER catalyst.^[^ ^28^ ^]^ Furthermore, inspired by the biomimetic molecular compounds with a hetero‐cubane architecture of a general formula (Ni/Co_4−*x*_Zn_*x*_O_4_; *x = *1–3), we synthesized novel oxide materials to investigate their electrocatalytic behavior during OER. By the solvothermal decomposition of a heterobimetallic Co_4−*x*_Zn_*x*_O_4_ dipyridyl diol precursor (*x* = 1–3) in benzylamine, we attained ZnO:M (M = Co, Ni) materials which were active toward (electro)chemical OER. The as‐synthesized ZnO:Co material with an exceptionally high Co content showed considerably low overpotential during OER in alkaline media. Its remarkable activity and stability was attributed to the unique active structure consisting of self‐supported crystalline ZnO:Co particles with an amorphous overlayer of hydroxylated CoZn that formed in situ at the applied potential.^[^ ^77^ ^]^

**Figure 4:**
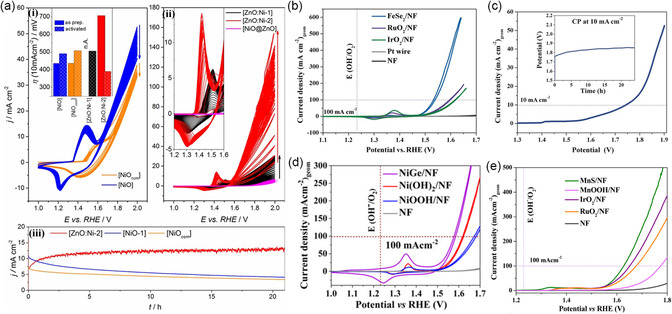
Electrochemical oxygen evolution reaction (OER) and overall water‐splitting (OWS) activity of SSP‐derived electrocatalysts. a) i) Cyclic voltammetry (CV) cycling of NiO and NiO_com_ (inset: required overpotential to reach 10 mA cm^−2^ current density), ii) CV cycling of different ZnO:Ni along with ZnO@NiO, and iii) stability tests of Ni‐substituted ZnO nanocrystallites (ZnO:Ni) prepared from Ni_4–*x*_Zn_*x*_O_4_ (*x* = 1–3), along with other control samples. ZnO: Ni‐2 shows better OER activity, which is further compared with NiO‐1 and commercially available (NiO_com_). a) Reproduced with permission.^[^ ^124^ ^]^ Copyright 2017, Wiley‐VCH. b) OER CV curves of FeSe_2_ along with state‐of‐the‐art noble‐metal‐based catalysts. c) OWS curve of FeSe_2_, recorded at 5 mV s^−1^ scan rate in 1 m KOH (inset: long‐term stability of the catalysts). b,c) Reproduced with permission.^[^ ^83^ ^]^ Copyright 2017, Wiley‐VCH. d) Catalytic performances of MnS and reference materials on NF. Reproduced with permission.^[^ ^14^ ^]^ Copyright 2022, Elsevier. e) Catalytic OER performances of NiGe along with other Ni‐based reference samples. Reproduced with permission.^[^ ^45^ ^]^ Copyright 2021, Wiley‐VCH.

Similarly, the ZnO:Ni material from the heterobimetallic Ni_4−*x*_Zn_*x*_O_4_ (*x* = 1–3) system transformed into a core–shell structure of a disordered mixture of *γ*‐NiOOH/*α*‐Ni(OH)_2_‐like phases on the surface by the dissolution of Zn^II^ from the ZnO:Ni (pre)catalyst into the electrolyte which was responsible for the activation and high OER performance in the basic electrolyte, with a high surface area, and was not observed in a similarly prepared NiO, highlighting the significance of the (pre)catalyst material for efficient water oxidation (**Figure** **4**a).^[^ ^124^ ^]^ In a series of studies, we used the benign *β*‐diketiminato ligand to synthesize all‐ferric molecular precursors to attain ultrasmall nanoparticles via the hot‐injection method as active materials in water‐splitting.^[^ 21, 83, 84 ^]^ The molecular [2Fe–2Se] and the [Fe_2_P_3_] cluster resulted in a FeSe_2_ and a FeP phase after the decomposition process, both served as electrode materials in alkaline OER, HER, and OWS with splendid activities **(**Figure 4b,c, **Table** **1**).^[^ 83, 84 ^]^ The [2Fe–2As] cluster resulted in a FeAs phase with outstanding activity in alkaline OER electrocatalysis.^[^ ^21^ ^]^ All prepared materials stood out with an enhanced electrochemical performance that was accounted to their extremely small and uniformly distributed particle size, their high surface area with enriched surface active sites, and their remarkably low resistivity. A comparative study on amorphous and crystalline CoP derived from a [Co_2_P_4_] cluster by two different thermolytic methods revealed that amorphous CoP is more active for HER, OER, and OWS than its crystalline counterpart. The amorphous CoP was obtained by the hot‐injection method, while the crystalline counterpart was attained by pyrolysis of the SSP in a quartz tube under an inert atmosphere. Although both phases showed similar transformation behavior, the change in the amorphous phase was more intense. The transformation into an active cobalt oxy(hydroxide)/oxide–enriched phase because of P dissolution as phosphate from the pre‐catalyst was higher in the amorphous phase than the crystalline. Further diverse morphologies, a higher surface area, and better electronic characteristics of the amorphous CoP compared to the crystalline phase contributed to the higher activity.^[^ ^125^ ^]^ Similarly, we conducted studies on several sulfide‐based materials by making use of the versatile 2‐mercaptopyridine as a supporting ligand to synthesize copper, manganese, and nickel‐based SSPs for material synthesis. The dinuclear copper(I) complex [{(PyHS)_2_Cu^I^(PyS}_2_](OTf)_2_, with a square planar {Cu^I^_2_S_2_} core, was transformed via hot injection into a crystalline Cu_9_S_5_ nanostructure. The in situ formed overlayer of CuO acted as active sites for the efficient OER catalysis while the highly conductive Cu_9_S_5_ core promoted fast charge transport between the catalytic‐active sites and substrate.^[^ ^48^ ^]^ The newly synthesized [Mn(PyHS)_6_](OTf)_2_ was transformed under hot‐injection conditions into a crystalline MnS phase at relatively mild conditions. The as‐prepared material showed excellent OER performance and long‐term durability in comparison to other reference materials (Figure 4d). Unexpectedly, the MnS transforms completely into the *β*‐Mn^III^OOH phase alkaline conditions at applied potentials which is retained even after prolonged catalytic potentials. Usually, this phase transforms further into a layered birnessite‐type *δ*‐MnO_2_ as observed in most oxidic or non‐oxidic Mn‐based materials; however, in this case, the *β*‐Mn^III^OOH was rather stable, further emphasizing the importance of 1 SSPs.^[^ ^14^ ^]^ Most recently, we designed a nickel (II) complex, [Ni^II^(PyHS)_4_][Otf]_2_ with a square‐planar {Ni^II^S_4_} core.^[^ ^46^ ^]^ Following a simple, single‐step synthetic protocol, the novel Ni^II^‐SSP could be easily transformed into nanostructured NiS using the hot‐injection method at relatively mild conditions. The SSP‐derived NiS proved to be a highly active (pre)catalyst for OER with low overpotential, and charge‐transfer resistance, smaller Tafel slope value, as well as better intrinsic properties when compared to reference materials. After electrocatalytic OER, we unveiled by in situ Raman spectroscopy, transmission electron microscopy (TEM), and X‐ray photoelectron spectroscopy (XPS) that the NiS was transformed to a carbonate‐intercalated *γ‐*NiOOH phase. The intercalation of CO_3_^2−^ anions between the formed [NiO_6_] layers of *γ*‐NiOOH enhanced the activity and acted as an active center for the catalyst. Benefitting from the SSP approach to design more active (pre)catalysts and to show their potential applications, we further applied the SSP‐derived NiS for selective oxidation of alcohol, aldehyde, and amine‐containing substrates with high yield and faradaic efficiency that was not possible to achieve with traditionally synthesized NiS (Table 1).^[^ ^46^ ^]^ Similar observations have been also made when we synthesized intermetallic NiGe (pre)catalysts from a xanthene‐based bis(germylene*)*–Ni complex. The as‐prepared NiGe showed ultrasmall particles sizes of 2 nm transformed during alkaline OER into the highly active *γ*‐NiOOH phase that acted as an active structure (Figure 4e). The clearly observed large interplanar spacing in the *γ*‐NiOOH structure provides the ionic intercalation of OH^−^/CO_3_^2−^, from dissolved CO_2_ in the electrolyte, which is favorable for the OER and further reflected in higher electrochemical‐active surface area values.^[^ 45, 46 ^]^ Additionally, we have lately prepared a highly OER‐active intermetallic crystalline manganese nitride (Mn_3_N_2_) through ammonolysis of manganocene [(C_5_H_5_)_2_Mn] as the SSP at 700 °C for 12 h using a tube furnace. The superior activity and durability of the electro(pre)catalyst were attributed to the transformation of surface manganese sites of Mn_3_N_2_ into an amorphous active MnO_*x*_. The obtained core–shell structure with the intact metallic Mn_3_N_2_ core increased the charge transfer from the active catalyst surface to the electrode substrates when compared to solely MnO_*x*_‐based catalysts.^[^ ^44^ ^]^ The results of all these studies clearly show that the overall predesign and the elemental composition in the desired material can be easily adjusted by suitable precursor synthesis and decomposition techniques. Therefore, the enhancement of its activity toward HER, OER, as well as OWS greatly depends on these choices to alter the physical, chemical, and electronic properties in the final (pre)catalyst material.

**Table 1:** Comparison of electrochemical OER and HER activities of SSP derived and conventionally synthesized materials. All the reported data measured in 1 m KOH (note, the direct comparison is difficult as the amount of catalyst loading, the particle size and surface area, *i*R compensation, substrate, reaction conditions, as well as the transformation pathways under operating conditions) can differ

	Material	Synthesis method	Activity	References
OER	NiS	SSP	*η*_10_ = 255 ± 2 mV	[46]
		Hydrothermal	*η*_10_ = 330 mV	[134]
	FeP	SSP	*η*_10_ = 227 mV	[84]
		Thermal annealing	*η*_10_ = 1.47 V	[135]
HER		SSP	*η*_−10_ = 166 mV	[84]
	FeP	Flux synthesis	*η*_−10 [010 facet]_ = 209 mV	[136]
		Hydrothermal and phosphorylation	*η*_−100_ = 193 mV	[137]

Along with superior activities, the SSP‐derived materials also showed long‐term stability under water‐splitting conditions, making them suitable for practical applications. For example, under the OER current density of 10 mA cm^−2^, NiGe showed remarkable stability for over 3 weeks.^[^ ^45^ ^]^ Long‐term OER stabilities were also exhibited by Mn_3_N_2_ for 180 h at 10 mA cm^−2^,^[^ ^44^ ^]^ CuCo_2_S_4_ for 40 h at 1.53 V,^[^ ^126^ ^]^ Co‐substituted ZnO for 6 h at 0.8 V_Ag/AgCl._^[^ ^77^ ^]^ Moreover, NiS exhibited stable performance under a high OER current density of 500 mA cm^−2^ for 10 h.^[^ ^46^ ^]^ Several SSP‐derived materials also showed long‐term stability for HER such as Mo_3+2*x*_Si_3_C_0.6_ for 35 h at −10 mA cm^−2^,^[^ ^114^ ^]^ NiPt_3_@NiS for 8 days at −0.012 V^[^ ^28^ ^]^ and NiSe for 60 h at −100 mA cm^−2^.^[^ ^99^ ^]^ In addition, NiSe displayed long‐term stability for OER and OWS for 60 h (at 100 mA cm^−2^) and for 12 h (at 50 mA cm^−2^), respectively.^[^ ^99^ ^]^ Furthermore, remarkable OWS stabilities were also observed for Fe_2_P_3_ (for 14 days at 10 mA cm^−2^),^[^ ^84^ ^]^ amorphous CoP (for 5 days at 10 mA cm^−2^),^[^ ^125^ ^]^ FeMnP (for 75 h at 1.60 V),^[^ ^100^ ^]^ and Bi_2_Se_3_ (for 20 h at 2.02 V).^[^ ^78^ ^]^ All these results suggested the superior stability of the SSP‐derived materials.

## 6 Post‐Electrochemical Characterization to Catch the Active Phases

**Figure 5:**
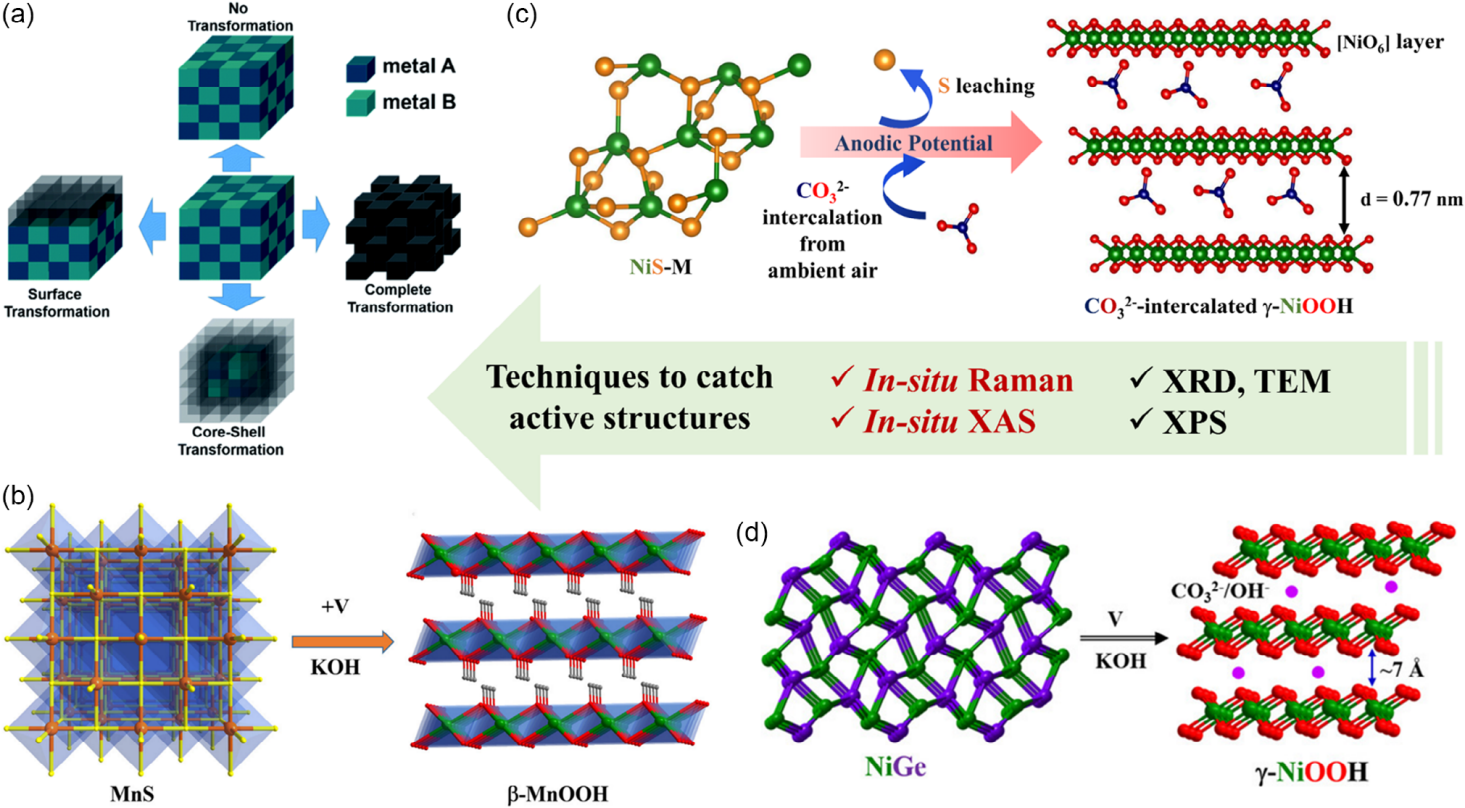
a) Possible transformation pathways of a (pre)catalyst into an active catalyst during catalytic OER and HER conditions. Reproduced with permission.^[^ ^61^ ^]^ Copyright 2021, Royal Society of Chemistry. b) Schematic representation of *β*‐MnOOH‐active phase formation under applied potential (in 1 m KOH), from molecularly derived MnS (pre)catalyst. Reproduced with permission.^[^ ^14^ ^]^ Copyright 2022, Elsevier. c) Crystal structure of NiS and electrochemically generated CO_3_^2−^‐intercalated active *γ*‐NiOOH phase during OER (in 1 m KOH electrolyte). Reproduced with permission.^[^ ^46^ ^]^ Copyright 2023, Wiley‐VCH. d) Crystal structure of molecular‐derived NiGe and electrochemically generated active phase (*γ‐*NiOOH) with ionic intercalation of OH^−^/CO_3_^2−^ during OER in an alkaline KOH. Reproduced with permission.^[^ ^45^ ^]^ Copyright 2021, Wiley‐VCH.

As discussed earlier, most of these SSP‐derived intermetallic, pnictide, and chalcogenide materials behave as (pre)catalysts for the electrochemical processes (OER, HER, OWS) under applied potentials and reconstruct into the actual active catalysts.^[^ 21, 45, 46, 77, 88, 100 ^]^ As shown in **Figure** **5**, the possible pathways for electrocatalyst reconstructions have already been discussed earlier, which lead to the formation of amorphous or crystalline, high surface area, and metal‐rich active structures.^[^ 52, 61 ^]^ Nevertheless, a detailed analysis of post‐electrochemical measurements of materials is required to obtain an in‐depth understanding of the reconstruction process, and their chemical composition, phase, morphology, and structural and electronic properties of the real active sites for catalysis.^[^ 45, 78, 127 ^]^ This would enable a thorough understanding of the structure–property relationships for the rational design of materials, with optimized properties, as highly efficient, stable, and cheap electrocatalysts for practical applications.^[^ 78, 127 ^]^ Several ex situ and in situ diffraction, microscopic, spectroscopic, and analytical characterization techniques have been adapted in this regard, which have proven to be highly efficient tools to reveal the true active structure of the catalysts.^[^ ^21^ ^]^ Some important and commonly applied characterization techniques which reveal the true active structures of OER, and HER will be discussed in the following sections.

### 6.1 Ex Situ Characterization Techniques

The preliminary examination of any material post OER/ HER electrochemical measurements is usually performed by powder X‐ray diffraction (pXRD), scanning electron microscopy (SEM), and energy‐dispersive X‐ray spectroscopy (EDX), to understand the phase (amorphous or crystalline), morphology, and percentage distribution of elements (or composition), respectively, of the transformed active structure.^[^ ^78^ ^]^ Such post‐electrocatalytic ex situ characterizations are usually carried out directly with the recovered as‐deposited working electrodes and/or by scratching off the materials from the recovered electrodes.^[^ ^83^ ^]^ Furthermore, the composition of the transformed phase can also be determined from the inductively coupled plasma atomic emission spectroscopy (ICP‐AES).^[^ ^94^ ^]^ In‐depth understanding of the nanostructure evolution of the (pre)catalysts can be obtained from TEM, high‐resolution TEM (HR‐TEM), and selected‐area electron diffraction (SAED).^[^ ^45^ ^]^ These methods paint a clear picture about the formation of near‐surface, core–shell, or completely transformed phases, the presence of heterostructures, nanocrystalline domains, expanded lattices and defects, etc.^[^ ^45^ ^]^ The transformation of the Mn_3_N_2_ (pre)catalyst for OER into an amorphous MnO_*x*_ shell and Mn_3_N_2_ core, or the transformation of the surface NiS phase of the NiPt_3_@NiS (pre)catalyst for HER, or the complete transformation of the NiGe (pre)catalyst for OER into a NiOOH‐active structure was all confirmed by the above ex situ techniques.^[^ 28, 44, 45 ^]^ Furthermore, XPS, a highly surface sensitive technique, is adapted to garner accurate information about the surface valence states, that is, the species present on the surface of the material and their oxidation states and ratios.^[^ ^99^ ^]^ For example, the XPS of the MnS (pre)catalyst post‐OER reveals the presence of a Mn^III^ peak in the Mn 2p spectrum, corresponding to the *β*‐Mn^III^OOH‐active structure.^[^ ^14^ ^]^ Similarly, the Ni^III^ peaks in the Ni 2p spectrum of the NiS (pre)catalyst after OER corresponds to the *γ*‐Ni^III^OOH‐active structure.^[^ ^46^ ^]^ Therefore, XPS is a highly efficient technique to reveal the active species involved in catalysis, when supported by other advanced in situ techniques such as Raman spectroscopy and X‐ray absorption spectroscopy (XAS).^[^ ^21^ ^]^ In addition to that, Fourier‐transform infrared spectroscopy (FTIR) is also an effective tool to reveal some information about the surface composition of the transformed phase.^[^ 45, 128 ^]^

### 6.2 In situ Characterization Techniques

**Figure 6:**
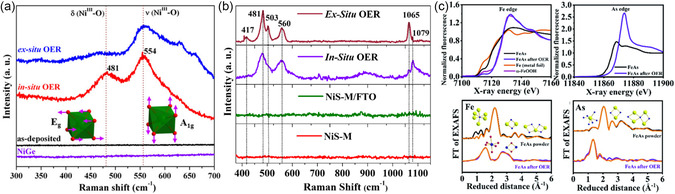
In situ and ex situ Raman spectroscopy. a) SSP‐derived NiGe, where the stretching (A_1g_) and bending (E_g_) vibrational modes of *γ*‐NiOOH is represented by green octahedra. Reproduced with permission.^[^ ^45^ ^]^ Copyright 2021, Wiley‐VCH. b) SSP‐derived NiS. The band at ≈1065 cm^−1^ characteristic signal of carbonate (CO_3_^2−^) intercalation. Reproduced with permission.^[^ ^46^ ^]^ Copyright 2023, Wiley‐VCH. c) X‐ray absorption near‐edge structure (XANES) spectra (top) and X‐ray absorption fine structure (EXAFS) spectra (bottom) of SSP‐derived FeAs. In the figure, blue, yellow, and red spheres represent Fe, As, and O atoms, respectively. Reproduced with permission.^[^ ^21^ ^]^ Copyright 2020, The Royal Society of Chemistry.

Advanced in situ or operando characterizations are extremely useful techniques to uncover the structural evolution, true active structure, active sites of a catalyst, and the reaction mechanisms under applied OER/ HER conditions. In situ/operando studies involve pairing the characterization instruments directly with the working cell, or freeze‐quenching the samples (in liquid N_2_, −196 °C) during OER/HER conditions, also called as quasi in situ techniques.^[^ 29, 45 ^]^ In this regard, in situ (resonance) Raman spectroscopy is an extremely sensitive and useful tool to identify the composition of the active phase present on the surface and the presence of any surface‐adsorbed or intercalated ions, or doped elements. Moreover, the potential‐dependent in situ Raman spectroscopy is used to establish the reaction mechanisms of catalysis. For example, the in situ Raman bands observed in the close region of ≈480 and ≈560 cm^−1^ for the stretching *δ*(Ni^III^–O) and bending *ν*(Ni^III^–O) vibrational modes, confirmed the formation of *γ*‐NiOOH‐active phase from NiGe and NiS (pre)catalysts and an additional band at 1065 cm^−1^ confirmed the intercalation of carbonate anion into the [NiO_6_] layers, evolving from NiS (pre)catalyst (**Figures** **6**a,b).^[^ 45, 46 ^]^ In the case of the NiS (pre)catalyst, we also observed other bands at 410 and 503 cm^−1^ corresponding to the Ni–O species and at 1079 cm^−1^ corresponding to active oxygen species. Furthermore, another often used highly advanced technique is the XAS, including X‐ray absorption near‐edge structure (XANES) and extended X‐ray absorption fine structure (EXAFS) analyses, which reveals the coordination of the metal centers (e.g., tetrahedral, octahedral, etc.) and their in situ oxidation states, the number of coordinatively unsaturated sites or edge sites, linkage of the unit cells (edge or corner sharing) for crystalline phases, etc.^[^ ^79^ ^]^ As shown in Figure 6c, the XANES spectra of FeAs were shifted toward a higher energy after OER, which suggested that FeAs oxidized to the +3 state of Fe^III^O_*x*_H_*y*_. Furthermore, the complete leaching out of As from the FeAs helped to the formation of a two‐line ferrihydrite‐active phase under alkaline OER conditions. The post‐OER EXAFS spectra revealed that the edge‐sharing FeO_6_ octahedron in the two‐line ferrihydrite induced more defects in edge/sites, which act as the active centers during OER. Several other characterization techniques also exist such as UV–vis spectroscopy, atomic force microscopy (AFM), focused‐ion beam, etc., and in situ XRD, XPS, TEM, wide‐angle X‐ray scattering, etc., as well as theoretical studies such as density‐functional theory, which are also often used by materials chemists to extract valuable information about the reconstructions during OER/HER.

## 7 Conclusion and Outlook

**Figure 7:**
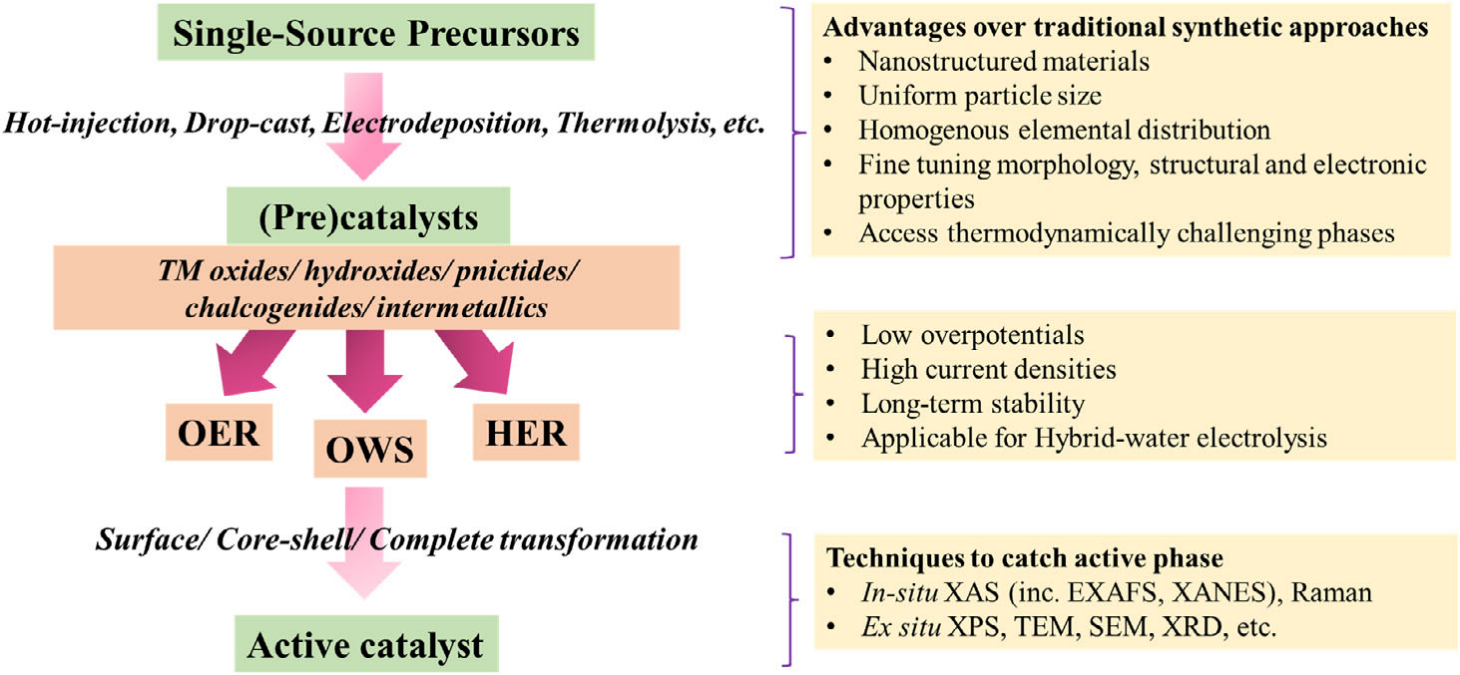
Flowchart summarizing the development of water‐splitting electrocatalysts from SSPs, advantages of the SSP approach, and the adopted characterization techniques to catch the active phases.

We have summarized the recent advancements in the single‐source molecular precursor approach toward the low temperature and structurally and electronically well‐controlled synthesis of nanomaterials as highly efficient and stable electro(pre)catalysts for OER, HER, and OWS. We have discussed the importance of water‐splitting in developing a carbon‐neutral economy, the fundamentals of water‐splitting, and the criteria for developing competitive electrocatalysts. Furthermore, we focused on the advantages of the low‐temperature single source over traditional high‐temperature methods in the development of superior materials with improved control over the composition, morphology, elemental distribution, particle size, and electronic properties of the material. By stating several important examples from the literature, it has been shown that the rationally designed suitable molecular complexes can be transformed into distinct oxide, carbide, pnictide, chalcogenide, and intermetallic phases by adopting various synthetic strategies. Most of these SSP‐derived phases are in fact (pre)catalysts, which under applied OER/HER condition, transform into an active phase like the oxides and (oxy)hydroxides. In‐depth information about the reconstruction, active structure, active species participating in catalysis, surface composition, intercalation, adsorption, reaction mechanism, etc., can be obtained from various ex situ and in situ techniques, which have also been discussed briefly in this review (**Figure** **7**).

However, the key challenges for synthesizing catalytic materials via the SSP approach are the time‐consuming synthesis of expensive molecular precursors, carbon contamination of the materials from the decomposed ligand, volatility, low vapor pressure, stoichiometry of the precursor not being preserved in the material, poor yields, poor atom economy, etc.^[^ 129, 130 ^]^ To design high‐performance electrocatalysts toward OER, HER, and OWS via the SSP approach, the principles that need to be followed include first a precursor screening, wherein complexes (reported in the Cambridge Structural Database) supported by similar ligands can be grouped together, and a systematic variation in the metal and nonmetal components in the complexes can be followed.^[^ ^71^ ^]^ The stoichiometry, phase, and particle size of the materials, which depend on the mechanism, rate of decomposition, and the respective phase diagrams, can also be fine‐tuned by modulating the ligand scaffolds, metal coordination environment, operating temperatures, and solvents, etc. Therefore, by optimizing these factors, materials with desired stoichiometry, phase purity, and nanosized particles with high surface areas can be achieved, leading to high‐electrocatalytic activities.^[^ ^71^ ^]^

In addition to OER, HER, and OWS, the scope of these SSP‐derived materials can also be extended toward hybrid water electrolysis, paired and seawater^[^ ^68^ ^]^ electrolysis. In hybrid water electrolysis, either OER or HER is replaced by an organic oxygenation and hydrogenation reaction, while H_2_ and O_2_ are produced at the counter electrode, respectively.^[^ ^131^ ^]^ In contrast, in paired electrolysis, an organic oxygenation reaction at the anode is paired with a hydrogenation reaction with a cathode.^[^ ^132^ ^]^ As mentioned earlier, our recent report on the SSP‐derived NiS phase catalyzed selectively 5‐hydroxymethylfurfural (HMF), benzyl alcohol, and benzylamine with high efficiencies.^[^ ^46^ ^]^ This application for this field has not been explored much so far but has a huge potential in the future and would enable the application of molecularly derived nanomaterials for a wide range of energy as well as chemical applications.^[^ ^133^ ^]^ Therefore, the SSP approach could indeed trigger the development of new electro‐(pre)catalysts for the catalysis industry in general and narrow the gap between molecular and materials chemistry as well as academic electrocatalytic research and industrial requirements.

## Conflict of Interest

The authors declare no conflict of interest.

## References

[1] N. S. Lewis , D. G. Nocera , Proc. Natl. Acad. Sci. USA 2006, 103, 15729. 17043226 10.1073/pnas.0603395103 PMC1635072

[2] L. Schlapbach , A. Züttel , Nature 2001, 414, 353. 11713542 10.1038/35104634

[3] T. J. Meyer , Nature 2008, 451, 778. 18273008 10.1038/451778a

[4] M. K. Debe , Nature 2012, 486, 43. 22678278 10.1038/nature11115

[5] A. Buttler , H. Spliethoff , Renew. Sustainable Energy Rev. 2018, 82, 2440.

[6] PWC: Green Hydrogen Economy - Predicted Development of Tomorrow , https://www.pwc.com/gx/en/industries/energy-utilities-resources/future-energy/green-hydrogen-cost.html (accessed: November 2022).

[7] Z. P. Ifkovits , J. M. Evans , M. C. Meier , K. M. Papadantonakis , N. S. Lewis , Energy Environ. Sci. 2021, 14, 4740.

[8] H. A. Miller , K. Bouzek , J. Hnat , S. Loos , C. I. Bernäcker , T. Weißgärber , L. Röntzsch , J. Meier-Haack , Sustainable Energy Fuels 2020, 4, 2114.

[9] S. Shiva Kumar , V. Himabindu , Mater. Sci. Energy Technol. 2019, 2, 442.

[10] D. Xu , M. B. Stevens , M. R. Cosby , S. Z. Oener , A. M. Smith , L. J. Enman , K. E. Ayers , C. B. Capuano , J. N. Renner , N. Danilovic , Y. Li , H. Wang , Q. Zhang , S. W. Boettcher , ACS Catal. 2019, 9, 7.

[11] C. C. L. McCrory , S. Jung , J. C. Peters , T. F. Jaramillo , J. Am. Chem. Soc. 2013, 135, 16977. 24171402 10.1021/ja407115p

[12] I. Ledezma-Yanez , W. D. Z. Wallace , P. Sebastián-Pascual , V. Climent , J. M. Feliu , M. T. M. Koper , Nat. Energy 2017, 2, 17031.

[13] J. N. Hausmann , R. Beltrán-Suito , S. Mebs , V. Hlukhyy , T. F. Fässler , H. Dau , M. Driess , P. W. Menezes , Adv. Mater. 2021, 33, 2008823. 34048605 10.1002/adma.202008823 PMC11468827

[14] C. Walter , S. Kalra , R. Beltrán-Suito , M. Schwarze , P. W. Menezes , M. Driess , Mater. Today Chem. 2022, 24, 100905.

[15] J. N. Hausmann , S. Mebs , H. Dau , M. Driess , P. W. Menezes , Adv. Mater. 2022, 34, 2207494. 10.1002/adma.202207494 36189873

[16] J. S. Kim , B. Kim , H. Kim , K. Kang , Adv. Energy Mater. 2018, 8, 1702774.

[17] Z. Guo , W. Ye , X. Fang , J. Wan , Y. Ye , Y. Dong , D. Cao , D. Yan , Inorg. Chem. Front. 2019, 6, 687.

[18] J. N. Hausmann , S. Mebs , K. Laun , I. Zebger , H. Dau , P. W. Menezes , M. Driess , Energy Environ. Sci. 2020, 13, 3607.

[19] S. Anantharaj , S. R. Ede , K. Sakthikumar , K. Karthick , S. Mishra , S. Kundu , ACS Catal. 2016, 6, 8069.

[20] M. A. Mushtaq , A. Kumar , G. Yasin , M. Arif , M. Tabish , S. Ibraheem , X. Cai , W. Ye , X. Fang , A. Saad , J. Zhao , S. Ji , D. Yan , Appl. Catal. B Environ. 2022, 317, 121711.

[21] R. Beltrán-Suito , V. Forstner , J. N. Hausmann , S. Mebs , J. Schmidt , I. Zaharieva , K. Laun , I. Zebger , H. Dau , P. W. Menezes , M. Driess , Chem. Sci. 2020, 11, 11834. 34123210 10.1039/d0sc04384b PMC8162750

[22] P. W. Menezes , A. Indra , C. Das , C. Walter , C. Göbel , V. Gutkin , D. Schmeiβer , M. Driess , ACS Catal. 2017, 7, 103.

[23] M. Zeng , H. Wang , C. Zhao , J. Wei , K. Qi , W. Wang , X. Bai , ChemCatChem 2016, 8, 708.

[24] S. Gupta , M. K. Patel , A. Miotello , N. Patel , Adv. Funct. Mater. 2020, 30, 1906481.

[25] P. W. Menezes , C. Walter , B. Chakraborty , J. N. Hausmann , I. Zaharieva , A. Frick , E. Hauff , H. Dau , M. Driess , Adv. Mater. 2021, 33, 2004098. 33491823 10.1002/adma.202004098 PMC11468780

[26] L. Reith , J. N. Hausmann , S. Mebs , I. Mondal , H. Dau , M. Driess , P. W. Menezes , Adv. Energy Mater. 2023, 13, 2203886.

[27] H. Vrubel , X. Hu , Angew. Chem., Int. Ed. 2012, 51, 12703. 10.1002/anie.201207111 23143996

[28] C. Panda , P. W. Menezes , S. Yao , J. Schmidt , C. Walter , J. N. Hausmann , M. Driess , J. Am. Chem. Soc. 2019, 141, 13306. 31411876 10.1021/jacs.9b06530

[29] I. Mondal , J. N. Hausmann , G. Vijaykumar , S. Mebs , H. Dau , M. Driess , P. W. Menezes , Adv. Energy Mater. 2022, 12, 2200269.

[30] J. N. Hausmann , R. A. Khalaniya , C. Das , I. Remy-Speckmann , S. Berendts , A. V. Shevelkov , M. Driess , P. W. Menezes , Chem. Commun. 2021, 57, 2184. 10.1039/d0cc08035g 33527109

[31] H. Yang , J. N. Hausmann , V. Hlukhyy , T. Braun , K. Laun , I. Zebger , M. Driess , P. W. Menezes , ChemCatChem 2022, 14, e202200293.

[32] B. Chakraborty , R. Beltrán-Suito , J. N. Hausmann , S. Garai , M. Driess , P. W. Menezes , Adv. Energy Mater. 2020, 10, 2001377.

[33] C.-P. Wang , Y. Feng , H. Sun , Y. Wang , J. Yin , Z. Yao , X.-H. Bu , J. Zhu , ACS Catal. 2021, 11, 7132.

[34] S. Lyu , C. Guo , J. Wang , Z. Li , B. Yang , L. Lei , L. Wang , J. Xiao , T. Zhang , Y. Hou , Nat Commun 2022, 13, 6171. 36257963 10.1038/s41467-022-33847-z PMC9579180

[35] W. Ye , Y. Yang , X. Fang , M. Arif , X. Chen , D. Yan , ACS Sustainable Chem. Eng. 2019, 7, 18085.

[36] M. Liu , S. Liu , C.-X. Cui , Q. Miao , Y. He , X. Li , Q. Xu , G. Zeng , Angew. Chem., Int. Ed. 2022, 61, e202213522. 10.1002/anie.202213522 36240790

[37] S. Mondal , B. Mohanty , M. Nurhuda , S. Dalapati , R. Jana , M. Addicoat , A. Datta , B. K. Jena , A. Bhaumik , ACS Catal. 2020, 10, 5623.

[38] S. Ghosh , A. Mondal , G. Tudu , S. Ghosh , H. V. S. R. M. Koppisetti , H. R. Inta , D. Saha , V. Mahalingam , ACS Sustainable Chem. Eng. 2022, 10, 7265.

[39] S. Ghosh , R. Jana , S. Ganguli , H. R. Inta , G. Tudu , H. V. S. R. M. Koppisetti , A. Datta , V. Mahalingam , Nanoscale Adv. 2021, 3, 3770. 36133027 10.1039/d1na00034a PMC9416859

[40] A. Indra , P. W. Menezes , I. Zaharieva , E. Baktash , J. Pfrommer , M. Schwarze , H. Dau , M. Driess , Angew. Chem., Int. Ed. 2013, 52, 13206. 10.1002/anie.201307543 24174384

[41] A. Indra , P. W. Menezes , F. Schuster , M. Driess , J. Photochem. Photobiol. B Biol. 2015, 152, 156. 10.1016/j.jphotobiol.2014.11.012 25542875

[42] Y. Xue , B. Huang , Y. Yi , Y. Guo , Z. Zuo , Y. Li , Z. Jia , H. Liu , Y. Li , Nat. Commun. 2018, 9, 1460. 29654234 10.1038/s41467-018-03896-4 PMC5899097

[43] C. Panda , P. W. Menezes , M. Driess , Angew. Chem., Int. Ed. 2018, 57, 11130. 10.1002/anie.201803673 29733547

[44] C. Walter , P. W. Menezes , S. Orthmann , J. Schuch , P. Connor , B. Kaiser , M. Lerch , M. Driess , Angew. Chem., Int. Ed. 2018, 57, 698. 10.1002/anie.201710460 29205790

[45] P. W. Menezes , S. Yao , R. Beltrán-Suito , J. N. Hausmann , P. V. Menezes , M. Driess , Angew. Chem., Int. Ed. 2021, 60, 4640. 10.1002/anie.202014331 PMC7986911 33169889

[46] S. Ghosh , B. Dasgupta , S. Kalra , M. L. P. Ashton , R. Yang , C. J. Kueppers , S. Gok , E. G. Alonso , J. Schmidt , K. Laun , I. Zebger , C. Walter , M. Driess , P. W. Menezes , Small 2023, 10.1002/smll.202206679. 36651137

[47] H. Lu , D. S. Wright , S. D. Pike , Chem. Commun. 2020, 56, 854. 10.1039/c9cc06258k 31859335

[48] B. Chakraborty , S. Kalra , R. Beltrán-Suito , C. Das , T. Hellmann , P. W. Menezes , M. Driess , Chem. Asian J. 2020, 15, 852. 32011083 10.1002/asia.202000022 PMC7155036

[49] J. Greeley , N. M. Markovic , Energy Environ. Sci. 2012, 5, 9246.

[50] L. Hui , Y. Xue , H. Yu , Y. Liu , Y. Fang , C. Xing , B. Huang , Y. Li , J. Am. Chem. Soc. 2019, 141, 10677. 31149825 10.1021/jacs.9b03004

[51] J. Durst , A. Siebel , C. Simon , F. Hasché , J. Herranz , H. A. Gasteiger , Energy Environ. Sci. 2014, 7, 2255.

[52] Z. Chen , H. Yang , Z. Kang , M. Driess , P. W. Menezes , Adv. Mater. 2022, 34, 2108432. 10.1002/adma.202108432 35104388

[53] H. Yang , M. Driess , P. W. Menezes , Adv. Energy Mater. 2021, 11, 2102074.

[54] J. N. Hausmann , P. V. Menezes , G. Vijaykumar , K. Laun , T. Diemant , I. Zebger , T. Jacob , M. Driess , P. W. Menezes , Adv. Energy Mater. 2022, 12, 2202098.

[55] W. Li , D. Xiong , X. Gao , L. Liu , Chem. Commun. 2019, 55, 8744. 10.1039/c9cc02845e 31268066

[56] Z. Yan , H. Liu , Z. Hao , M. Yu , X. Chen , J. Chen , Chem. Sci. 2020, 11, 10614. 34094316 10.1039/d0sc01532f PMC8162381

[57] C. Wei , Z. J. Xu , Small Methods 2018, 2, 1800168.

[58] J. Zhu , L. Hu , P. Zhao , L. Y. S. Lee , K.-Y. Wong , Chem. Rev. 2020, 120, 851. 31657904 10.1021/acs.chemrev.9b00248

[59] R. Gao , J. Zhu , D. Yan , Nanoscale 2021, 13, 13593. 34477633 10.1039/d1nr03409j

[60] R. Gao , D. Yan , Adv. Energy Mater. 2020, 10, 1900954.

[61] C. Walter , P. W. Menezes , M. Driess , Chem. Sci. 2021, 12, 8603. 34257861 10.1039/d1sc01901e PMC8246119

[62] Z. Chen , H. Yang , S. Mebs , H. Dau , M. Driess , Z. Wang , Z. Kang , P. W. Menezes , Adv. Mater. 2023, 35, 2208337. 10.1002/adma.202208337 36528302

[63] G. Ertl , H. Knozinger , J. Weitkamp , Handbook of Heterogeneous Catalysis, Wiley-VCH, Weinheim, Germany 1999, p. 28.

[64] Y. Zeng , M. Zhao , Z. Huang , W. Zhu , J. Zheng , Q. Jiang , Z. Wang , H. Liang , Adv. Energy Mater. 2022, 12, 2201713.

[65] X. Liu , R. Guo , K. Ni , F. Xia , C. Niu , B. Wen , J. Meng , P. Wu , J. Wu , X. Wu , L. Mai , Adv. Mater. 2020, 32, 2001136. 10.1002/adma.202001136 32876959

[66] H. Yang , P. V. Menezes , G. Dai , G. Vijaykumar , Z. Chen , M. Al-Shakran , T. Jacob , M. Driess , P. W. Menezes , Appl. Catal. B Environ. 2023, 324, 122249.

[67] Y. Gao , Y. Xue , L. Qi , C. Xing , X. Zheng , F. He , Y. Li , Nat. Commun. 2022, 13, 5227. 36064713 10.1038/s41467-022-32937-2 PMC9445080

[68] Y. Gao , Y. Xue , F. He , Y. Li , Proc. Natl. Acad. Sci. USA 2022, 119, e2206946119. 36037378 10.1073/pnas.2206946119 PMC9457402

[69] H. Zhang , A. W. Maijenburg , X. Li , S. L. Schweizer , R. B. Wehrspohn , Adv. Funct. Mater. 2020, 30, 2003261.

[70] X. Liu , J. Meng , J. Zhu , M. Huang , B. Wen , R. Guo , L. Mai , Adv. Mater. 2021, 33, 2007344. 10.1002/adma.202007344 34050565

[71] C. L. Daniels , D. L. Mendivelso-Perez , B. A. Rosales , D. You , S. Sahu , J. S. Jones , E. A. Smith , F. P. Gabbaï , J. Vela , ACS Omega 2019, 4, 5197. 31459692 10.1021/acsomega.9b00088 PMC6648806

[72] S. Polarz , A. V. Orlov , M. W. E. van den Berg , M. Driess , Angew. Chem., Int. Ed. 2005, 44, 7892. 10.1002/anie.200501212 16299820

[73] X. Zhu , T. Jin , C. Tian , C. Lu , X. Liu , M. Zeng , X. Zhuang , S. Yang , L. He , H. Liu , S. Dai , Adv. Mater. 2017, 29, 1704091. 10.1002/adma.201704091 29068542

[74] B. K. Barman , K. K. Nanda , Dalton Trans. 2016, 45, 6352. 26999042 10.1039/c6dt00536e

[75] S. M. Al-Zuraiji , T. Benkó , K. Frey , Z. Kerner , J. S. Pap , Catalysts 2021, 11, 577.

[76] L. Tong , W. Wu , K. Kuepper , A. Scheurer , K. Meyer , ChemSusChem 2018, 11, 2752. 29883067 10.1002/cssc.201800971

[77] J. Pfrommer , M. Lublow , A. Azarpira , C. Göbel , M. Lücke , A. Steigert , M. Pogrzeba , P. W. Menezes , A. Fischer , T. Schedel-Niedrig , M. Driess , Angew. Chem., Int. Ed. 2014, 53, 5183. 10.1002/anie.201400243 24777630

[78] S. Razzaque , M. D. Khan , M. Aamir , M. Sohail , S. Bhoyate , R. K. Gupta , M. Sher , J. Akhtar , N. Revaprasadu , Inorg. Chem. 2021, 60, 1449. 33464045 10.1021/acs.inorgchem.0c02668 PMC8716079

[79] A. Singh , S. L. Y. Chang , R. K. Hocking , U. Bach , L. Spiccia , Energy Environ. Sci. 2013, 6, 579.

[80] O. M. Yaghi , M. O’Keeffe , N. W. Ockwig , H. K. Chae , M. Eddaoudi , J. Kim , Nature 2003, 423, 705. 12802325 10.1038/nature01650

[81] Y. Yang , Y. Yang , Y. Liu , S. Zhao , Z. Tang , Small Sci. 2021, 1, 2100015. 10.1002/smsc.202100015 PMC11936017 40212851

[82] S. Li , Y. Gao , N. Li , L. Ge , X. Bu , P. Feng , Energy Environ. Sci. 2021, 14, 1897.

[83] C. Panda , P. W. Menezes , C. Walter , S. Yao , M. E. Miehlich , V. Gutkin , K. Meyer , M. Driess , Angew. Chem., Int. Ed. 2017, 56, 10506. 10.1002/anie.201706196 28678439

[84] S. Yao , V. Forstner , P. W. Menezes , C. Panda , S. Mebs , E. M. Zolnhofer , M. E. Miehlich , T. Szilvási , N. Ashok Kumar , M. Haumann , K. Meyer , H. Grützmacher , M. Driess , Chem. Sci. 2018, 9, 8590. 30568784 10.1039/c8sc03407a PMC6253717

[85] G. Siddiqi , V. Mougel , C. Copéret , Inorg. Chem. 2016, 55, 4026. 27064051 10.1021/acs.inorgchem.6b00341

[86] A. Ziani , T. Shinagawa , L. Stegenburga , K. Takanabe , ACS Appl. Mater. Interfaces 2016, 8, 32376. 27813407 10.1021/acsami.6b12006

[87] J. R. Swierk , T. D. Tilley , J. Electrochem. Soc. 2018, 165, H3028.

[88] J. J. Stracke , R. G. Finke , J. Am. Chem. Soc. 2011, 133, 14872. 21894961 10.1021/ja205569j

[89] J. J. Stracke , R. G. Finke , ACS Catal. 2013, 3, 1209.

[90] Y.-H. Lai , C.-Y. Lin , Y. Lv , T. C. King , A. Steiner , N. M. Muresan , L. Gan , D. S. Wright , E. Reisner , Chem. Commun. 2013, 49, 4331. 10.1039/c2cc34934e 22918295

[91] Y.-H. Lai , D. W. Palm , E. Reisner , Adv. Energy Mater. 2015, 5, 1501668.

[92] V. Riesgo-Gonzalez , S. Bhattacharjee , X. Dong , D. S. Hall , V. Andrei , A. D. Bond , C. P. Grey , E. Reisner , D. S. Wright , Inorg. Chem. 2022, 61, 6223. 35412823 10.1021/acs.inorgchem.2c00403 PMC9098167

[93] D. A. Kuznetsov , D. V. Konev , S. A. Sokolov , I. V. Fedyanin , Chem. - Eur. J. 2018, 24, 13890. 30030924 10.1002/chem.201802632

[94] H. Chen , Y. Gao , L. Ye , Y. Yao , Y. Wei , X. Chen , Electrochim. Acta 2018, 283, 104.

[95] S. Suseno , C. C. L. McCrory , R. Tran , S. Gul , J. Yano , T. Agapie , Chem. - Eur. J. 2015, 21, 13420. 26246131 10.1002/chem.201501104 PMC4868073

[96] C. R. R. Gan , Z. Liu , S.-Q. Bai , S. W. Tay , X. Ge , J.-E. Wu , T. S. A. Hor , Inorg. Chem. Front. 2014, 1, 705.

[97] E. Anxolabéhère-Mallart , C. Costentin , M. Fournier , S. Nowak , M. Robert , J.-M. Savéant , J. Am. Chem. Soc. 2012, 134, 6104. 22458714 10.1021/ja301134e

[98] W.-L. Li , T.-W. Chiou , C.-H. Chen , Y.-J. Yu , L.-K. Chu , W.-F. Liaw , Dalton Trans. 2018, 47, 7128. 29756619 10.1039/c8dt00601f

[99] D. Chen , Y. Chen , W. Zhang , R. Cao , New J. Chem. 2021, 45, 351.

[100] Z. Zhao , D. E. Schipper , A. P. Leitner , H. Thirumalai , J.-H. Chen , L. Xie , F. Qin , M. K. Alam , L. C. Grabow , S. Chen , D. Wang , Z. Ren , Z. Wang , K. H. Whitmire , J. Bao , Nano Energy 2017, 39, 444.

[101] D. E. Schipper , Z. Zhao , A. P. Leitner , L. Xie , F. Qin , M. K. Alam , S. Chen , D. Wang , Z. Ren , Z. Wang , J. Bao , K. H. Whitmire , ACS Nano 2017, 11, 4051. 28333437 10.1021/acsnano.7b00704

[102] D. E. Schipper , Z. Zhao , H. Thirumalai , A. P. Leitner , S. L. Donaldson , A. Kumar , F. Qin , Z. Wang , L. C. Grabow , J. Bao , K. H. Whitmire , Chem. Mater. 2018, 30, 3588.

[103] N. Jiménez-Arévalo , F. Leardini , I. J. Ferrer , J. R. Ares , C. Sánchez , M. M. Saad Abdelnabi , M. G. Betti , C. Mariani , ACS Appl. Energy Mater. 2020, 3, 1922.

[104] A. Mishra , A. Mehta , S. Basu , N. P. Shetti , K. R. Reddy , T. M. Aminabhavi , Carbon N Y 2019, 149, 693.

[105] D. A. Kuznetsov , D. V. Konev , N. S. Komarova , A. M. Ionov , R. N. Mozhchil , I. V. Fedyanin , Chem. Commun. 2016, 52, 9255. 10.1039/c6cc04400j 27354324

[106] S. Shanmugam , A. Sivanantham , M. Matsunaga , U. Simon , T. Osaka , Electrochim. Acta 2019, 297, 749.

[107] J. Q. Zhao , D. Cai , J. Dai , M. Kurmoo , X. Peng , M. H. Zeng , Sci. Bull. 2019, 64, 1667. 10.1016/j.scib.2019.09.013 36659780

[108] R. Akram , M. D. Khan , C. Zequine , C. Zhao , R. K. Gupta , M. Akhtar , J. Akhtar , M. A. Malik , N. Revaprasadu , M. H. Bhatti , Mater. Sci. Semicond. Process 2020, 109, 104925.

[109] R. Zhang , P. A. Russo , M. Feist , P. Amsalem , N. Koch , N. Pinna , ACS Appl. Mater. Interfaces 2017, 9, 14013. 28357856 10.1021/acsami.7b01178

[110] Z. Liao , Y. Wang , Q. Wang , Y. Cheng , Z. Xiang , Appl. Catal. B Environ. 2019, 243, 204.

[111] H. Zhuang , Y. Xie , H. Tan , Y. Deng , Y. Li , G. Chen , Electrochim. Acta 2018, 262, 18.

[112] B. Sarkar , D. Das , K. K. Nanda , Green Chem. 2020, 22, 7884.

[113] Y. Chen , C. Tian , T. Jiang , C. Maheu , J. P. Hofmann , L. Molina-Luna , R. Riedel , Z. Yu , ChemPlusChem 2022, 87, e202200338. 36478656 10.1002/cplu.202200338

[114] Y. Feng , Z. Yu , J. Schuch , S. Tao , L. Wiehl , C. Fasel , W. Jaegermann , R. Riedel , J. Am. Ceram. Soc. 2020, 103, 508.

[115] Z. Yu , K. Mao , Y. Feng , J. Adv. Ceram. 2021, 10, 1338.

[116] Y.-Y. Chen , Y. Zhang , W.-J. Jiang , X. Zhang , Z. Dai , L.-J. Wan , J.-S. Hu , ACS Nano 2016, 10, 8851. 27617483 10.1021/acsnano.6b04725

[117] X. Xing , R. Liu , K. Cao , U. Kaiser , C. Streb , Chem. - Eur. J. 2019, 25, 11098. 31106936 10.1002/chem.201901400

[118] X. Xing , R. Liu , K. Cao , U. Kaiser , G. Zhang , C. Streb , ACS Appl. Mater. Interfaces 2018, 10, 44511. 30508370 10.1021/acsami.8b16578

[119] L. Qiao , A. Zhu , W. Zeng , R. Dong , P. Tan , Z. Ding , P. Gao , S. Wang , J. Pan , J. Mater. Chem. A 2020, 8, 2453.

[120] E. T. Nguyen , I. A. Bertini , A. J. Ritz , R. A. Lazenby , K. Mao , J. R. McBride , A. V. Mattia , J. E. Kuszynski , S. F. Wenzel , S. D. Bennett , G. F. Strouse , Inorg. Chem. 2022, 61, 13836. 36007248 10.1021/acs.inorgchem.2c01713

[121] G. E. Ayom , M. D. Khan , T. Ingsel , W. Lin , R. K. Gupta , S. J. Zamisa , W. E. Zyl , N. Revaprasadu , Chem. - Eur. J. 2020, 26, 2693. 31773811 10.1002/chem.201904583

[122] P. Oswal , K. Sood , S. Singh , A. Arora , A. Bahuguna , S. Purohit , A. Kumar , Dalton Trans. 2022, 51, 6537. 35441183 10.1039/d2dt00347c

[123] M. Muddassir , A. Alarifi , N. A. Y. Abduh , W. S. Saeed , A. M. Karami , M. Afzal , Materials (Basel) 2022, 16, 299. 36614637 10.3390/ma16010299 PMC9822453

[124] J. Pfrommer , A. Azarpira , A. Steigert , K. Olech , P. W. Menezes , R. F. Duarte , X. Liao , R. G. Wilks , M. Bär , T. Schedel-Niedrig , M. Driess , ChemCatChem 2017, 9, 672.

[125] R. Beltrán-Suito , P. W. Menezes , M. Driess , J. Mater. Chem. A 2019, 7, 15749.

[126] R. Zhang , Z. Hu , S. Cheng , W. Ke , T. Ning , J. Wu , X. Fu , G. Zhu , Inorg. Chem. 2021, 60, 6721. 33861926 10.1021/acs.inorgchem.1c00545

[127] Z. Li , B. Li , C. Yang , S. Lin , Q. Pang , P. Shen , Appl. Surf. Sci. 2019, 491, 723.

[128] L. Zeng , X. Cui , J. Zhang , W. Huang , L. Chen , C. Wei , J. Shi , Electrochim. Acta 2018, 275, 218.

[129] P. Marchand , C. J. Carmalt , Coord. Chem. Rev. 2013, 257, 3202.

[130] C. E. Knapp , C. J. Carmalt , Chem. Soc. Rev. 2016, 45, 1036. 26446057 10.1039/c5cs00651a

[131] G. Chen , X. Li , X. Feng , Angew. Chem., Int. Ed. 2022, 61, e202209014. 10.1002/anie.202209014 PMC9826310 35849025

[132] P. Li , T. Zhang , M. A. Mushtaq , S. Wu , X. Xiang , D. Yan , Chem. Rec. 2021, 21, 841. 33656241 10.1002/tcr.202000186

[133] Á. Vass , B. Endrődi , C. Janáky , Curr. Opin. Electrochem. 2021, 25, 100621.

[134] A. Mehmood , G. Rahman , A. U. H. A. Shah , O. Joo , S. A. Mian , Energy Fuels 2021, 35, 6868.

[135] R. Zhang , C. Zhang , W. Chen , J. Mater. Chem. A 2016, 4, 18723.

[136] B. Owens-Baird , J. P. S. Sousa , Y. Ziouani , D. Y. Petrovykh , N. A. Zarkevich , D. D. Johnson , Y. V. Kolen'ko , K. Kovnir , Chem. Sci. 2020, 11, 5007. 34122957 10.1039/d0sc00676a PMC8159208

[137] Y. Yan , X. R. Shi , M. Miao , T. He , Z. H. Dong , K. Zhan , J. H. Yang , B. Zhao , B. Y. Xia , Nano Res. 2018, 11, 3537.

